# Pneumoperitoneum Caused by a Ruptured Splenic Abscess Mimicking Gastrointestinal Perforation: A Case Report

**DOI:** 10.70352/scrj.cr.24-0098

**Published:** 2025-04-25

**Authors:** Naoki Kawahara, Mitsuaki Kojima, Koji Morishita

**Affiliations:** Trauma and Acute Critical Care Centre, Institute of Science Tokyo Hospital, Bunkyo-ku, Tokyo, Japan

**Keywords:** splenic abscess, pneumoperitoneum, open abdominal management, emergency exploratory laparotomy

## Abstract

**INTRODUCTION:**

Splenic abscess is a rare but potentially life-threatening condition that can rupture, leading to pneumoperitoneum and symptoms that mimic gastrointestinal perforation in rare cases. This can significantly complicate accurate diagnosis and prompt treatment. A splenic abscess can become life-threatening by rupturing, which may cause diffuse peritonitis or sepsis.

**CASE PRESENTATION:**

A 68-year-old man with uncontrolled diabetes presented with fever, chills, and abdominal pain. Initial evaluation at a previous hospital, including computed tomography (CT), suggested a lower gastrointestinal perforation, leading to his transfer to our facility. CT revealed a non-enhancing lesion with gas in the spleen and free intraperitoneal air; however, there was no clear evidence of gastrointestinal perforation. An emergency exploratory laparotomy was performed, which revealed purulent ascites and a ruptured splenic abscess without any gastrointestinal perforation. After thorough lavage to eliminate contamination, open abdominal management was initiated owing to a need for catecholamine support and an inability to completely rule out the possibility of gastrointestinal perforation. A second-look laparotomy confirmed that there was no further contamination or gastrointestinal tract perforation. Blood and abscess cultures revealed *Escherichia coli*, leading us to initiate targeted antibiotic therapy. The patient recovered successfully and was discharged on postoperative day 40 without any recurrence. Ruptured splenic abscess with pneumoperitoneum is rare and poses significant diagnostic challenges, particularly in patients with diabetes, owing to its clinical similarity to gastrointestinal perforation. This study highlights the utility of exploratory laparotomy and staged open abdominal management when gastrointestinal perforation cannot be ruled out.

**CONCLUSIONS:**

Physicians should consider ruptured splenic abscesses in patients with pneumoperitoneum, particularly those with diabetes. Exploratory laparotomy with staged open abdominal management may represent an effective approach that facilitates safe monitoring and targeted treatment, thereby reducing the risk of fatal complications.

## Abbreviations

CT
: computed tomography
IE
: infective endocarditis
OAM
: open abdominal management
PCD
: percutaneous drainage
POD
: postoperative day
SOFA
: sequential organ failure assessment

## INTRODUCTION

Splenic abscess is a rare infectious condition with an estimated incidence ranging between 0.14% and 0.7%, according to several autopsy studies.^1,2)^ Although the mortality rate associated with this condition has decreased in recent years, it remains significant, with a few reports indicating a mortality rate as high as 14%.^2,3)^ Moreover, splenic abscess is recognized as a potential cause of non-traumatic splenic rupture.^4)^ Herein, we report a patient with splenic abscess rupture that presented with pneumoperitoneum, which complicated diagnosis because of its similarity to gastrointestinal perforation.

## CASE PRESENTATION

**Fig. 1:**
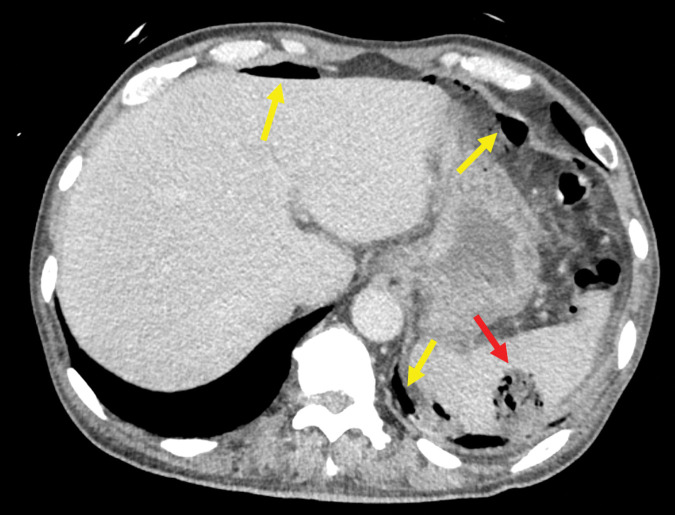
Contrast-enhanced computed tomography findings: A non-enhancing area containing gas was observed in the upper pole of the spleen (red arrow), along with free intraperitoneal gas that primarily surrounded the spleen (yellow arrow).

The patient was a 68-year-old man with a history of diabetes for which he was not receiving regular follow-up care. He developed chills and rigors while working, prompting him to call emergency services. He was initially diagnosed with suspected lower gastrointestinal perforation at another hospital before being transferred to our facility for further evaluation. The patient’s vital signs upon arrival were as follows: Glasgow Coma Scale score of E4V5M6, body temperature of 39.5°C, heart rate of 132 bpm, blood pressure of 114/72 mmHg, respiratory rate of 28 breaths/min, and SpO_2_ of 93% on room air. Physical examination revealed tenderness and rebound pain in the mid-abdomen. Laboratory tests revealed an elevated inflammatory response (white blood cell: 21900/µL, C-reactive protein: 13.89 mg/dL) without apparent signs of liver dysfunction (aspartate aminotransferase 52 U/L, alanine transaminase 90 U/L, γ-glutamyl transpeptidase 121 U/L, and total bilirubin 1.1 mg/dL) or kidney dysfunction (blood urea nitrogen 15.4 mg/dL and creatinine 0.69 mg/dL). Coagulation function was normal (platelet count 564000/μL, prothrombin time-international normalized ratio 1.06, activated partial thromboplastin time 25.7 seconds and fibrinogen 639 mg/dL), and the patient’s hemoglobin A1c level was 10.3%. Venous blood gas analysis revealed pH 7.494, pO_2_ 41.6 mmHg, pCO_2_ 35.0 mmHg, lactate 1.4 mmol/L, and HCO_3_^–^ 26.7 mmol/L—thus indicating no evidence of acidosis. Based on these vital signs and blood test results upon arrival, the patient’s sequential organ failure assessment (SOFA) score was considered zero. Contrast-enhanced computed tomography (CT) revealed a non-enhancing lesion with scattered gas in the upper pole of the spleen and free intraperitoneal air predominantly around the spleen (**Fig. 1**). No definitive evidence of gastrointestinal perforation was observed, but scattered non-enhancing areas were present in both kidneys. Electrocardiography revealed atrial fibrillation. Given these findings, the differential diagnoses included a ruptured splenic abscess, multiple renal abscesses, and possible gastrointestinal perforation. Blood cultures were obtained, and intravenous administration of meropenem was initiated. Despite the lack of a clear perforation site on imaging, the presence of significant free gas led us to believe that this possibility could not be ruled out, prompting us to perform an emergency exploratory laparotomy. Because the patient had a shock index greater than 1.0, indicating hemodynamic instability, exploratory laparotomy was preferred over diagnostic laparoscopy.

With the patient under general anesthesia, a midline incision was made in the upper abdomen. Upon entering the peritoneal cavity, we observed purulent ascites without fecal odor. Examination of the stomach, small intestine, and colon revealed no evidence of perforation. We observed that the patient’s omentum was adhered to the spleen. Following separation, we identified an indentation at the upper splenic pole surrounded by purulent material. We sent samples of ascitic fluid and the purulent material for culturing and irrigated the peritoneal cavity with saline solution. During surgery, the patient required norepinephrine infusion (0.05–0.1 µg/kg/min) to maintain a mean arterial pressure of 65 mmHg. Given the ongoing need for catecholamine support and the inability to completely rule out gastrointestinal perforation, open abdominal management (OAM) was selected.

Postoperatively, the patient’s SOFA score increased to 6; however, his hemodynamics stabilized and the need for catecholamine support had resolved by postoperative day 1 (POD 1). We performed a second-look laparotomy on POD 2. Only a small volume of purulent ascites was present, and reexamination of the stomach, small intestine, and colon once again showed no signs of perforation. Three drains were placed: 1 each beneath the left and right sides of the diaphragm and 1 in the rectovesical pouch. The patient’s condition was improving by the time of the second-look procedure, leading us to conclude that infection control had been adequately achieved through lavage and drainage alone. Therefore, we chose not to perform a splenectomy and closed the abdomen after placing the drains. Cultures of the blood, ascitic fluid, and purulent material samples from the area near the upper splenic pole grew *Escherichia coli*, prompting us to switch the patient’s antibiotic regimen to cefmetazole. The patient’s condition stabilized, and he was extubated on POD 3.

Considering that the initial CT findings suggested multiple renal abscesses, we investigated the possibility of infectious endocarditis. Transthoracic echocardiography on POD 3 revealed no vegetation. We initiated anticoagulation therapy owing to ongoing atrial fibrillation. Repeat blood cultures conducted on POD 4 tested positive for *E. coli* on POD 5, leading us to switch the patient to ceftriaxone based on susceptibility testing results. By POD 6, the patient’s SOFA score had returned to zero. Further blood culture results on POD 7 were negative. The drain in the rectovesical pouch was removed on POD 7, and the right subphrenic drain was removed on POD 12. On POD 11, a follow-up contrast-enhanced CT was performed to evaluate the patient’s condition. This revealed an air-containing fluid collection at the upper pole of the spleen, similar to the findings we observed upon the patient’s admission, which suggested a potential residual splenic abscess. Small non-enhancing areas were scattered in both kidneys, indicating the persistence of the multiple renal abscesses that were previously identified on admission. No other intra-abdominal abscesses were detected. A transesophageal echocardiography performed on POD 17 did not reveal any vegetation, suggesting that infective endocarditis (IE) was unlikely. However, given the presence of bilateral renal abscesses, this possibility could not be completely ruled out. Furthermore, the findings of residual splenic and renal abscesses prompted us to administer antibiotics for 28 days after the final negative blood culture, to ensure comprehensive infection control. Another follow-up CT performed on POD 27 revealed that both the splenic abscess and the bilateral renal abscesses had shrunk in size, indicating a positive response to the ongoing antibiotic therapy. After 4 weeks of negative blood cultures, ceftriaxone was discontinued on POD 35. The left subphrenic drain was gradually removed, with final removal occurring on POD 38. The patient was discharged to his home on POD 40. When writing this report, he has reported no symptom recurrence since his discharge.

## DISCUSSION

This patient presented with pneumoperitoneum, which was most likely caused by a ruptured splenic abscess. The symptoms associated with splenic abscess are often nonspecific, including fever, abdominal pain, and left-upper quadrant pain.^5–7)^ An overview of the management of splenic abscesses is presented in **Table 1**.^5–16)^ Gastrointestinal perforation is the most common cause of pneumoperitoneum.^17,18)^ Splenic abscess represents an exceedingly rare cause of this condition, and even fewer cases result in rupture.^1,2,4)^ Reports of ruptured splenic abscess with pneumoperitoneum in the literature are limited to a handful of case studies,^17–21)^ making it challenging to differentiate from gastrointestinal perforation as a potential cause. To provide additional context, we conducted a comprehensive literature search of the PubMed database using the query (“splenic abscess” OR “spleen abscess”) AND (“rupture” OR “perforation”) AND (“pneumoperitoneum” OR “free air”). This search identified 13 case reports. After excluding 1 case involving a canine patient and 2 cases of secondary splenic abscesses caused by cancer invasion, we selected 10 cases for analysis. We summarized these 10 cases in **Table 2**,^16,18–20,22–27)^ extracting details such as patient demographics, clinical presentations, imaging findings, treatment strategies, and outcomes. For some of the cases, this level of detail was either not provided in the original manuscript or could not be accessed—instances are denoted as “Not specified” in the table. These cases highlight the variability in clinical management, ranging from splenectomy to conservative approaches such as lavage and drainage, depending on each patient’s condition and disease severity.

**Table 1:** Treatment process for splenic abscess

**Diagnosis and initial assessment**
*Clinical features*
Splenic abscess often presents with fever, left upper quadrant pain, and leukocytosis—but these symptoms lack specificity.^5–7)^
*Imaging*
Computed tomography (CT):
CT imaging is highly sensitive (92%–96%) and specific (90%–95%) for diagnosing splenic abscesses.^8)^
Ultrasound:
Ultrasound has been reported to have a sensitivity of 70% and a specificity of 96%.^9)^
**Treatment options**
Antibiotics alone:
Small abscesses (defined as <4 cm in 1 study^6)^) are considered treatable using antibiotic therapy alone. While earlier studies suggested poor outcomes with antibiotic therapy alone, more recent ones have reported favorable outcomes.^6,10)^
Percutaneous drainage (PCD):
PCD is particularly effective for patients who are considered poor candidates for surgery; and most appropriate when the abscess is unilocular or bilocular, has a well-defined wall without internal septations, and contains sufficiently liquid material for drainage.^11,12)^
PCD has been reported to represent a bridge to definitive treatment in high-risk patients.^13)^
Splenectomy:
Splenectomy, which has been recognized as the gold standard treatment, represents the most effective and definitive option, despite its higher mortality rate. It is therefore reserved for cases where conservative treatment (e.g., antibiotics with or without PCD) fails, or when abscesses are recurrent, ruptured with peritonitis, are multiloculated, or are associated with thick-septa and necrosis.^13–16)^

**Table 2:** Summary of reported cases of ruptured splenic abscesses with pneumoperitoneum

Author (publication year)	Patient demographic (age/sex)	Underlying diseases	Clinical presentation	Treatment	Outcome
Puhakka KB, et al. (1997)^23)^	91/Male	Colonic cancer, melanosarcoma, etc.	Diffuse abdominal pain	Splenectomy	Death
Rege SA, et al. (2001)^24)^	30/Male	Not specified	Not specified	Splenectomy	Recovered
Ishigami K, et al. (2003)^18)^	55/Male	HIV-positive, an abdominal aortic occlusion, etc.	Diffuse abdominal pain	Splenectomy	Not specified
Braat MN, et al. (2009)^22)^	55/Male	Ulcerative colitis	Upper abdominal pain and fever	Lavage	Recovered
van Nunspeet L, et al. (2013)^25)^	78/Male	Mitral valve insufficiency, diverticulosis, etc.	Upper left abdominal pain	Splenectomy	Recovered
Narra RK, et al. (2015)^20)^	48/Male	Diabetes	Fever, headache, and left hypochondrial pain	Lavage	Recovered
Peña-Ros E, et al. (2015)^16)^	55/Male	Diabetes, ischemic heart disease, and tongue cancer	Abdominal pain and fever	Splenectomy	Recovered
Barrón-Reyes JE, et al. (2017)^26)^	50/Male	Diabetes, cirrhosis, chronic renal failure, etc.	Back pain and vomiting	Splenectomy	Recovered
Agarwal N, et al. (2019)^19)^	62/Female	None	Upper abdominal pain and fever	Splenectomy	Recovered
Zarei E, et al. (2023)^27)^	14/Female	Diabetes and vesicourethral reflux	Left upper abdominal pain and fever	Splenectomy	Recovered

In this case, preoperative CT revealed free extraluminal gas, leading us to consider gastrointestinal perforation and perform an exploratory laparotomy. Intraoperatively, we observed no gastrointestinal perforation, suggesting that the pneumoperitoneum was caused by a splenic abscess rupture. However, given the risk of missing the perforation site, OAM was performed to ensure safe monitoring.

During second-look surgery, we observed no new contamination in the abdominal cavity, and reexamination of the gastrointestinal tract confirmed the absence of perforation. Based on these findings, we concluded that the pneumoperitoneum resulted from a ruptured splenic abscess and completed the abdominal closure. Although OAM can increase invasiveness, similar to prolonged intubation, this approach allowed us to safely exclude the potentially fatal risk of missed gastrointestinal perforation.

In our case, the patient’s hemodynamic status stabilized after lavage and drainage without the need for splenectomy. While splenectomy is commonly considered the gold standard for treating ruptured splenic abscesses, it is an invasive procedure that is associated with significant risks such as infection.^28)^ Therefore, avoiding splenectomy, if feasible, was a priority in this case. Several case reports in the literature, including some involving pneumoperitoneum, have reported that lavage and drainage alone successfully treated the condition.^20,22)^ These cases suggest that in certain situations, such as when the patient’s condition is stable and adequate infection control can be achieved, a conservative approach may be preferable.

The decision to perform staged surgery in this case was influenced by the emergence of a need for catecholamine support, indicating hemodynamic instability, during the initial procedure—as well as our priority to rule out the possibility of gastrointestinal perforation. The 2-day interval between the first and second surgery allowed for careful monitoring of the patient’s clinical course and ensured that hemodynamic stability was confirmed before abdominal closure. Although a shorter interval may have been feasible, this timing allowed us to avoid splenectomy by confirming infection control and hemodynamic stabilization through the second-look procedure. Had the surgery been completed during the initial operation, the patient’s instability would likely have warranted splenectomy, which we aimed to avoid because of its invasiveness.

Although the effectiveness of OAM in splenic abscess cases has not been established, some reports suggest that the use of OAM in cases of splenic abscess with peritonitis may be associated with reduced mortality.^29)^ The present case highlights the potential utility of this approach in terms of managing peritonitis caused by ruptured splenic abscesses, particularly in terms of achieving a balance between infection control and organ preservation. However, the limited number of reported cases wherein this approach was applied successfully emphasizes that further studies are warranted to clarify the indications, timing, and criteria for this approach.

Risk factors for splenic abscesses include trauma, splenic infarction, diabetes, HIV infection, malignancy, and chemotherapy-related immunosuppression in patients with cancer.^18,20)^ In this patient, untreated diabetes likely contributed as a risk factor. Common pathogens reported in patients with splenic abscess ruptures include *Streptococci*, *Staphylococcus*, *Klebsiella*, and *E. coli*.^18,20,21)^ In this study, *E.coli* was identified in the ascitic fluid, purulent material, and blood cultures, indicating that it was the causative agent. *E.coli* is known to cause gas-forming abscesses in the spleen and liver.^30,31)^ Along with *Klebsiella pneumoniae*, it is particularly associated with gas-forming infections in patients with diabetes.^31)^ In this study, gas-producing *E. coli* in the patient’s splenic abscess likely led to the release of free intraperitoneal gas upon its rupture.

Splenic abscesses can arise from various mechanisms: hematogenous spread in immunocompromised patients or those with IE; secondary hematogenous infection in spleens affected by splenic infarction; or direct invasion from adjacent organs, such as migrating pancreatic abscesses or those that move through perforations in the stomach or colon.^19)^ In our patient, the presence of untreated diabetes, multiple renal abscesses, and atrial fibrillation suggested a possible primary hematogenous or secondary infection caused by an infarction associated with atrial fibrillation. IE was considered; however, transthoracic and transesophageal echocardiography revealed no vegetation. Although the source of infection remained unclear, we followed the protocol for IE by administering antibiotics for 4 weeks after confirming negative blood cultures. When writing this report, the patient has shown no signs of splenic abscess recurrence since his discharge.

## CONCLUSIONS

Although splenic abscess ruptures are quite rare, they can nevertheless cause pneumoperitoneum. The combination of its rarity and nonspecific symptoms makes splenic abscess rupture difficult to differentiate from gastrointestinal perforation. When managing a splenic abscess rupture with pneumoperitoneum and considering the risk of gastrointestinal perforation, an exploratory laparotomy approach followed by staged surgery using OAM may comprise a valuable treatment option.

## ACKNOWLEDGMENTS

The authors thank the patient and his family, as well as the physicians, nurses, paramedics, and staff members who attended to this case.

## DECLARATIONS

### Funding

This study did not receive any specific grants from funding agencies in the public, commercial, or not-for-profit sectors.

### Authors’ contributions

NK and MK contributed to drafting the manuscript and the study design.

MK critically advised during the manuscript drafting.

All of the authors have read and approved the final submitted version of the manuscript.

### Availability of data and materials

Not applicable.

### Ethics approval and consent to participate

This study was conducted in accordance with the principles of the Declaration of Helsinki.

### Consent for publication

Informed consent for publication of the clinical details and images presented herein was obtained from the patient.

### Competing interests

The authors declare that they have no competing interests.

## References

[1] Westh H Reines E Skibsted L. Splenic abscesses: a review of 20 cases. Scand J Infect Dis 1990; 22: 569–73. 2259866 10.3109/00365549009027098

[2] Chang KC Chuah SK Changchien CS Clinical characteristics and prognostic factors of splenic abscess: a review of 67 cases in a single medical center of Taiwan. World J Gastroenterol 2006; 12: 460–4. 16489650 10.3748/wjg.v12.i3.460 PMC4066069

[3] Hung SK Ng CJ Kuo CF Comparison of the Mortality in Emergency Department Sepsis Score, Modified Early Warning Score, Rapid Emergency Medicine Score and Rapid Acute Physiology Score for predicting the outcomes of adult splenic abscess patients in the emergency department. PLoS One 2017; 12: e0187495. 29091954 10.1371/journal.pone.0187495 PMC5665602

[4] Bhullar AA Canders CP Rouhani A Splenic abscess leading to spontaneous splenic rupture. Emerg Care J. 2021; 17.

[5] Liang JT Lee PH Wang SM Splenic abscess: a diagnostic pitfall in the ED. Am J Emerg Med 1995; 13: 337–43. 7755833 10.1016/0735-6757(95)90215-5

[6] Lee MC Lee CM. Splenic abscess: an uncommon entity with potentially life-threatening evolution. Can J Infect Dis Med Microbiol 2018; 1: 8610657. 10.1155/2018/8610657 PMC5830950 29666665

[7] Lee WS Choi ST Kim KK. Splenic abscess: a single institution study and review of the literature. Yonsei Med J 2011; 52: 288–92. 21319348 10.3349/ymj.2011.52.2.288 PMC3051211

[8] Nieciecki M Kozuch M Czarniecki M How to diagnose splenic abscesses? Acta Gastroenterol Belg 2019; 82: 421–6. 31566331

[9] Li XZ Song J Sun Z Conventional ultrasound and contrast-enhanced ultrasound in the diagnosis of splenic diseases: a systematic review and meta-analysis. J Ultrasound Med 2020; 39: 1687–94. 32323353 10.1002/jum.15291

[10] Liu KY Shyr YM Su CH Splenic abscess: a changing trend in treatment. S Afr J Surg 2000; 38: 55–7. 11392197

[11] Zerem E Bergsland J. Ultrasound-guided percutaneous treatment of splenic abscesses: the significance in treatment of critically ill patients. World J Gastroenterol 2006; 12: 7341–5. 17143953 10.3748/wjg.v12.i45.7341 PMC4087495

[12] Gutama B Wothe JK Xiao M Splenectomy versus imaging-guided percutaneous drainage for splenic abscess: a systematic review and meta-analysis. Surg Infect (Larchmt) 2022; 23: 417–29. 35612434 10.1089/sur.2022.072 PMC9208856

[13] Gomes CA Júnior CS Coccolini F Splenectomy in non-traumatic diseases. Australas Med J 2018; 11: 295–304.

[14] Raju T Paruthy SB Mohan SK Splenic abscess presenting as a case of pneumoperitoneum: a rare presentation. Int Surg J 2021; 8: 2502–04.

[15] George P Ahmed A Maroli R Peritonitis secondary to ruptured splenic abscess: a grave complication of typhoid fever. Asian Pac J Trop Med 2012; 5: 1004–6. 23199723 10.1016/S1995-7645(12)60191-6

[16] Peña-Ros E Méndez-Martínez M Vicente-Ruiz M Neumoperitoneo por absceso esplénico: un reto diagnóstico. Reporte de un caso. [Pneumoperitoneum due to splenic abscess: a diagnostic challenge. Case Report]. Cir Cir 2015; 83: 433–7. (in Spanish) 26164134 10.1016/j.circir.2015.05.044

[17] Kaur G Singh T Goyal S Ruptured splenic abscess with pneumoperitoneum: a rare presentation. J Acute Care Surg 2023; 13: 138–40.

[18] Ishigami K Decker GT Bolton-Smith JA Ruptured splenic abscess: a cause of pneumoperitoneum in a patient with AIDS. Emerg Radiol 2003; 10: 163–5. 15290509 10.1007/s10140-003-0302-7

[19] Agarwal N Sharma A Garg G. Non-traumatic ruptured splenic abscess presenting with pneumoperitoneum in an immunocompetent patient: a diagnostic dilemma. BMJ Case Rep 2019; 12: e228961. 10.1136/bcr-2018-228961 PMC6506054 31068349

[20] Narra RK Jehendran MV. Ruptured splenic abscess causing pneumoperitoneum: a rare cause revisited. BMJ Case Rep 2015; 2015: bcr2014209055. 10.1136/bcr-2014-209055 PMC4368946 25795751

[21] Bauman Z Lim J. Pneumoperitoneum as a result of a ruptured splenic abscess. J Surg Case Rep 2013; 2013: rjt111. 24968441 10.1093/jscr/rjt111 PMC3888002

[22] Braat MN Hueting WE Hazebroek EJ. Pneumoperitoneum secondary to a ruptured splenic abscess. Intern Emerg Med 2009; 4: 349–51. 19415449 10.1007/s11739-009-0253-4

[23] Puhakka KB Boljanovic S. Ruptured splenic abscess as cause of pneumoperitoneum. Rofo 1997; 166: 273–4. 9134035 10.1055/s-2007-1015425

[24] Rege SA Philip U Quentin N Ruptured splenic abscess presenting as pneumoperitoneum. Indian J Gastroenterol 2001; 20: 246–7. 11817784

[25] van Nunspeet L Eddes EH de Noo ME. Uncommon cause of pneumoperitoneum. World J Gastrointest Surg 2013; 5: 329–31. 24392184 10.4240/wjgs.v5.i12.329 PMC3879417

[26] Barrón-Reyes JE Chávez-Galván JC Martínez-Peralta JA Rotura esplénica secundaria a absceso, causa poco común de neumoperitoneo. Reporte de un caso. [Splenic rupture secondary to abscess: rare cause of pneumoperitoneum. Case report]. Cir Cir 2017; 85(Suppl 1): 62–7; (in Spanish) 28027808 10.1016/j.circir.2016.10.021

[27] Zarei E Pour Mohammad A Vafadar M Ruptured splenic abscess as an extremely rare cause of pneumoperitoneum: a comprehensive review with a case report. Radiol Case Rep 2023; 18: 4380–3. 37929045 10.1016/j.radcr.2023.09.025 PMC10624761

[28] Thomsen RW Schoonen WM Farkas DK Risk for hospital contact with infection in patients with splenectomy: a population-based cohort study. Ann Intern Med 2009; 151: 546–55. 19841456 10.7326/0003-4819-151-8-200910200-00008

[29] Petersen S, Deder A Prause A Transverse vs. median laparotomy in peritonitis and staged lavage: a single center case series. GMS Ger Med Sci 2020; 18: Doc07. 32973421 10.3205/000283 PMC7492753

[30] Wang M Yang L Yang Y A rare emphysematous splenic infection caused by diabetes mellitus: a case report. Ann Palliat Med 2021; 10: 10091–4. 34628928 10.21037/apm-21-2097

[31] Kurasawa M Nishikido T Koike J Gas-forming liver abscess associated with rapid hemolysis in a diabetic patient. World J Diabetes 2014; 5: 224–9. 24748935 10.4239/wjd.v5.i2.224 PMC3990319

